# Complete Genome Sequence of *Staphylococcus aureus* Strain Wood 46

**DOI:** 10.1128/genomeA.00087-17

**Published:** 2017-03-30

**Authors:** Manasi Balachandran, Matthew C. Riley, David A. Bemis, Stephen A. Kania

**Affiliations:** Department of Biomedical and Diagnostic Sciences, University of Tennessee, Knoxville, Tennessee, USA

## Abstract

Here, we report the first complete genome sequence of the *Staphylococcus aureus* strain Wood 46. Wood 46 has played an important role in understanding the virulence and pathogenesis of *S. aureus* infections. This report will assist efforts in vaccine development against methicillin-resistant *S. aureus* (MRSA) infections.

## GENOME ANNOUNCEMENT

*Staphylococcus aureus* is a Gram-positive opportunistic pathogen that infects both humans and animals. It causes skin and soft tissue infections, and is also associated with septic arthritis, pneumonia, post-surgical and implant infections, osteomyelitis, septicemia, and toxic shock syndrome (1, 2). Methicillin-resistant *S. aureus* (MRSA) is an important concern in hospitals (HA-MRSA), communities (CA-MRSA), and among livestock (LA-MRSA) (2, 3). New vaccine discovery efforts rely on understanding the role of surface and/or secreted proteins in pathogenesis and infection (4, 5). The *S. aureus* Wood 46 strain was reported to be protein A deficient and assumed to be *spa* negative (6). It has also been shown to have reduced virulence in animal models of *S. aureus* infection (7–15). To date, the reason for reduced virulence and protein A expression in the Wood 46 strain remains unknown. The whole-genome sequence of this strain could provide insight into the genes, promoters, and regulatory regions involved in pathogenesis and virulence. This in turn could help researchers understand the role of specific virulence factors during infection and identify novel vaccine targets and/or develop novel strategies to combat MRSA infections.

The *S. aureus* Wood 46 strain was sourced from the American Type Culture Collection (ATCC 10832). DNA was sequenced using Illumina MiSeq (Illumina, Inc., USA). *De novo* assemblies were individually produced and merged using Geneious (version 9.1.6) and CLC Genomics Workbench (version 9.0). Automated annotation was performed using the NCBI Prokaryotic Genome Annotation Pipeline (http://ncbi.nlm.nih.gov/genome/annotation_prok).

A total of 3,985,114 high-quality reads resulted in >180-fold overall coverage. The genome is 2,824,597 bp with a 32.8% G+C content. The number of predicted coding sequences (CDS) and genes (RNAs) were 2,806 and 73, respectively. The Wood 46 genome shared 98% to 99% identity with other published *S. aureus* genomes. The bacterium is susceptible to amoxicillin-clavulanic acid, cefoxitin, cephalothin, chloramphenicol, clindamycin, erythromycin, gentamicin, marbofloxacin, oxacillin, tetracycline, and trimethroprim/sulfa, and resistant to ampicillin and cefpodoxime (16).

### Accession number(s).

The whole-genome shotgun project of the *S. aureus* strain Wood 46 has been deposited at DDBJ/ENA/GenBank under the accession number MTFQ00000000. The version described in this paper is MTFQ00000000.1.
